# IMPROVING THE PERFORMANCE OF COMMUNITY HEALTH WORKERS IN A BEHAVIORAL LIFESTYLE INTERVENTION FOR OLDER ADULTS

**DOI:** 10.1093/geroni/igac059.1845

**Published:** 2022-12-20

**Authors:** Xinran Liu, Yuxin Lin, Sara Baumann, Steven Albert

**Affiliations:** University of Pittsburgh, Pittsburgh, Pennsylvania, United States; Peking University Health Science Center, Beijing, Beijing, China (People's Republic); University of Pittsburgh, Pittsburgh, Pennsylvania, United States; University of Pittsburgh, Pittsburgh, Pennsylvania, United States

## Abstract

Community health workers (CHWs) play essential roles in implementing community-based behavioral lifestyle interventions. This qualitative analysis aimed to explore what program factors and CHW characteristics would help improve CHW performance in the Mobility and Vitality Lifestyle Program (MOVE UP), a community-based behavioral weight-management intervention for improving mobility among overweight or obese older adults. The MOVE UP program was implemented from January 2015 to June 2019 at 23 community-based sites in Pittsburgh, Pennsylvania. Trained and supported CHWs delivered 32 group sessions over 13 months. We collected data from semi-structured interviews with 21 CHWs and 14 community key informants (community site directors or coordinators); seven focus group discussions with participants from 9 sites, and 124 pieces of graduation advice provided by MOVE UP graduates for future participants; onsite CHW performance observation at 18 sites, and 124 meeting memos of support phone calls with CHWs. Interviews and focus groups were audio-recorded and transcribed verbatim. All data were thematically analyzed in a deductive approach, using the CHW generic logic model. Emerging themes were: Trained CHWs still require onsite support by experienced CHWs or training staff to deliver the first session; Participants need a Q&A session by specialists to address their questions beyond CHWs’ knowledge; Desirable CHW characteristics could increase CHWs’ relation and accessibility to participants, such as the same age group, similar weight loss experience, and living or working in the same community. These findings provide important insight as to recruiting and supporting CHWs in a community-based behavioral lifestyle intervention for older adults.

